# Genetic Engineering and Genome Editing Advances to Enhance Floral Attributes in Ornamental Plants: An Update

**DOI:** 10.3390/plants12233983

**Published:** 2023-11-27

**Authors:** Manjulatha Mekapogu, Hyun-Young Song, So-Hyeon Lim, Jae-A Jung

**Affiliations:** Floriculture Research Division, National Institute of Horticultural & Herbal Science, Rural Development Administration, Wanju 55365, Republic of Korea

**Keywords:** CRISPR/Cas9, ornamental attributes, gene editing, transgenics, ornamental plants

## Abstract

The ornamental horticulture industry is a highly dynamic and rapidly changing market. Constant development of novel cultivars with elite traits is essential to sustain competitiveness. Conventional breeding has been used to develop cultivars, which is often laborious. Biotechnological strategies such as genetic engineering have been crucial in manipulating and improving various beneficial traits that are technically not possible through cross-breeding. One such trait is the highly desired blue-colored flower in roses and chrysanthemums, which can be achieved through transgenic technology. Advances in genome sequencing platforms have enhanced the opportunities to access the whole genome sequence in various ornamentals, facilitating the dissection of the molecular genetics and regulatory controls of different traits. The recent advent of genome editing tools, including CRISPR/Cas9, has revolutionized plant breeding. CRISPR/Cas9-based gene editing offers efficient and highly precise trait modification, contributing to various beneficial advancements. Although genome editing in ornamentals is currently in its infancy, the recent increase in the availability of ornamental genome sequences provides a platform to extend the frontiers of future genome editing in ornamentals. Hence, this review depicts the implication of various commercially valuable ornamental attributes, and details the research attempts and achievements in enhancing floral attributes using genetic engineering and genome editing in ornamental plants.

## 1. Introduction

Ornamental plants possess the potential to enhance aesthetic beauty with their delightful blooms and natural charm. The major trait of ornamentals is ‘beauty’, as they provide visual delight with their colorful and diverse-shaped flowers, fruits and leaves. They are frequently used for cut flowers, potted plants, landscaping, gardening and floristry [1]. Floriculture is witnessing an increasing global demand with an enhanced availability of diverse ornamental species [2]. The floriculture sector has a remarkable influence on the horticultural industry, contributing a phenomenal turnover from different market sections including cut flowers, landscaping, potted plants, ornamental foliage, nursery plants and bulbous plants. Among these, cut flowers alone make up as much as one third of the global ornamental market value [3,4]. Popular cut flowers, including roses, chrysanthemums, tulips, carnations and lilies, possess crucial economic importance in floriculture due to their aesthetic significance. The global production value of the flower and ornamental market in 2022 is valued at USD 40.25 billion and is expected to increase to USD 43.91 billion in 2023. The turnover of top major ornamental plants during 2022 in the largest global flower auction, Royal FloraHolland, is provided in Table 1 [5]. Ornamental plant production has thus been emerging as a profitable sector around the globe. However, the strength to withstand global competitiveness depends on the constant availability of elite cultivars with novel as well as trendy phenotypes, floral color patterns and fragrance, and these traits in turn are consumer choice-based, highly dynamic traits [6]. Hence, the constant breeding and production of new ornamental cultivars are crucial demands for breeders.

Various breeding strategies have been employed to develop novel and improved ornamental plants. Classical breeding techniques including hybridization, double haploids, mutagenesis and polyploidization have played a key role in the production of novel varieties with better traits. Apart from these techniques being laborious and time-consuming, genetic variations appear at a lower frequency [7]. In addition, ornamental plant classical breeding encounters several drawbacks, such as male sterility, longer breeding cycles, limited gene pool availability, polyploidy and higher heterozygosity causing a complex inheritance of traits [3]. Mutation breeding involving chemical and radiation mutagenesis produces genome-wide random mutations enabling the expansion of genetic variations and diversity [8]. However, the random nature of mutations accompanied by the laborious screening of mutant plants with desired traits and the induction of chimeras with difficulties in phenotype inheritance are the major limitations of mutational breeding [9]. Alternatively, transgenic technology, which has the potential to control desired traits via genetic transformation by overexpressing and suppressing genes, has resulted in improvements in various commercially valuable traits like floral color, fragrance, flower longevity and biotic and abiotic stress tolerance in ornamental plants [10]. Compared with conventional cross-breeding, genetic engineering has better breeding efficiency, significantly shortens the breeding duration and possesses improved precision and control of target traits [11]. Genetic engineering is often hindered by the lack of availability of candidate gene resources and transformation protocols for several ornamental crops [12]. Although various ornamental plants have been transformed for different traits, only a few genetically modified ornamental cultivars, such as color-modified carnations, roses and petunias, have been commercialized in a few countries [13]. The commercialization of genetically modified plants is stringently regulated for biosafety and risk assessment reasons in various countries [14]. Recent advances in genome sequencing technologies, particularly NGS and multi-omic platforms, have been playing a key role in the breeding and development of novel cultivars. NGS has emerged as a powerful tool in deciphering the genome sequence information of various ornamental plants in recent years. An increased number of ornamental plant genomes are being sequenced to enable the unraveling of molecular and regulatory mechanisms and thus help improve ornamental breeding.

The recent advent of genome editing, with the potential of revolutionizing crop improvement, acts as a promising tool for improvement in the traits and breeding of new varieties [15]. Genome editing allows efficient manipulation of specific traits and genes by enabling the precise modifications of target DNA for the purposes of modifying their expression or silencing them [16]. This technology thus offers an efficient expedition of breeding for crop improvement by overcoming genetic barriers and challenges in ornamental plants. Genome editing includes different tools like zinc finger nucleases (ZFNs), transcriptional activator-like effector nucleases (TALENs) and the CRISPR/Cas9 system [17,18,19]. Both ZFNs and TALENs use DNA-binding proteins that are engineered and customized to target specific genome sequences [20]. Nevertheless, proper design and assembly of new ZFNs are required along with the laborious screening process [21]. In contrast, the design of TALENs, which are an alternative to ZFNs, has been easier compared with that of the latter. The recently emerged clustered regularly interspaced short palindromic repeats-CRISPR-associated 9 (CRISPR/Cas9) is a breakthrough genome editing tool that utilizes guide RNA to navigate the Cas9 enzyme to precisely cleave the DNA to induce mutations and thereby modify gene expression [22]. Genome editing is a futuristic technology that works towards significantly contributing to the enhancement of ornamental characteristics.

This review therefore presents an overview of recent advances in genetic engineering and genome editing applications in ornamental plants. We discuss the current status of research efforts for the enhancement of ornamental attributes in popular ornamental plants such as roses, chrysanthemums, carnations, lilies, gerberas, tulips, freesias and others using these powerful biotechnological tools.

## 2. General Mechanism of Gene Editing and Genetic Engineering

Sequence-specific nucleases (SSNs) that can induce mutations via additions, deletions or sequence alteration at a specific locus are used for gene editing [23]. These SSNs are majorly classified into ZFNs, TALENs, meganucleases and CRISPR/Cas9, which have been efficiently employed in genome editing. ZFNs are the hybrid proteins of engineered endonucleases and artificial fusion proteins connecting a zinc finger DNA-binding domain to a nonspecific DNA cleavage domain of the FokI restriction endonuclease [24]. An engineered ZFN constitutes ZFN monomers tagged to an 18–24 bp DNA sequence with a spacer [25]. The ZFN domain identifies the target DNA sequence and the FokI domain cleaves the DNA, inducing modifications [26]. TALENs are designed endonucleases that can introduce double-stranded breaks (DSBs) at target DNA sequences. Similar to ZFNs, TALENs constitute a DNA-binding domain typically derived from transcription activator-like effectors (TALEs) and a nuclease domain from the FokI endonuclease [27]. Multiple repeats of TALEs constitute the TALEN’s DNA-binding domain, with each repeat identifying a specific nucleotide in the target sequence [28], whereas the nuclease domain from the FokI endonuclease needs dimerization for DNA cleavage. TALENs are usually made in pairs that target one each of the DNA strands. After entering the host cell, the DNA-binding domain attaches to the target site, and the FokI domain dimerizes, forming a functional nuclease complex which further induces DSBs at the target site [29].

The CRISPR/Cas9 system is a breakthrough genome editing technology that has gained immense attention recently due to its higher efficiency and adaptability in the genetic manipulation of various organisms [30]. It was first identified and derived from the adaptive immune systems of bacteria and archaea. Bacteria’s mechanism of defense against viral infections via the CRISPR method was found to be effective in the precise editing of plant genomes [31]. The CRISPR/Cas9 system is composed of two major components: guide RNA (gRNA) and the Cas9 nuclease. Typically, the CRISPR system involves RNA–DNA binding, unlike ZFNs and TALENS, which depend on protein–DNA binding for target sequence specificity. The Cas9 nuclease component comprises a recognition domain that includes two RNA-binding domains and a protospacer adjacent motif (PAM) domain which enables binding to the target DNA [32]. The endonuclease activity of the nuclease domain of Cas9 cleaves the DNA at the target location with the help of HNH and RuvC-like nuclease domains within the Cas9 protein, resulting in DSBs at the target DNA sequence [33]. gRNA is a synthetic RNA that helps in the guiding of the Cas9 nuclease to the target site. gRNA is essentially made up of two components, CRISPR RNA (crRNA) and transactivating CRISPR RNA (tracrRNA). crRNA provides the information on the complementary sequence of the target DNA, whereas tracrRNA combines with crRNA, forming a complex to assist in the assembly and stabilization of Cas9-gRNA [16,34]. The gRNA complex therefore guides the Cas9 nuclease to the target site, and the Cas9 cleaves the DNA by inducing DSBs at the gRNA complimentary site sequence [35]. DNA repair mechanisms are triggered following the cleavage and the DSBs are repaired either via homology-directed repair (HDR), which uses the template DNA and repairs the DSBs, or alternatively via nonhomologous end joining (NHEJ), which produces the indels that can disrupt the genes [36].

Genetic engineering employs the transfer of candidate genes into plants via *Agrobacterium*-mediated genetic transformation, and various ornamental plants have been transformed using this method, leading to substantial advancements in the development of novel cultivars with desired traits. Bud regeneration is the major regeneration protocol used in ornamental species, with the leaf as a main explant; for bulbous plants, protocorm is used as an explant [10,37]. Regeneration protocols are often unavailable in woody ornamentals because of their recalcitrant nature. However, alternative methods such as somatic callus induction and somatic embryo development have been used for woody species [38]. Laborious tissue culturing for regeneration could be bypassed through the floral dipping method of *Agrobacterium*-mediated gene transformation, which was successfully applied in various ornamental plants [39,40]. Nevertheless, transformation efficiency, adaptability and stability of the transformed plants are challenging steps which vary in different ornamental species and need to be established specifically for each cultivar [41].

**Figure 1:**
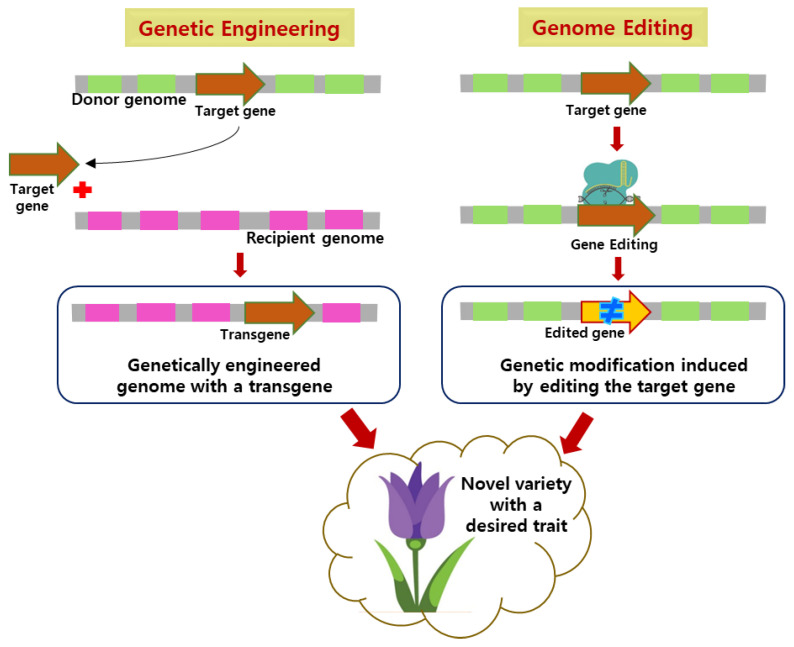
Image representing the development of novel varieties with improved traits via genetic engineering and genome editing.

Potential challenges associated with genome editing and genetic engineering are as follows. Molecular and genomic studies are highly challenging in ornamental plants, which is the primary hindrance to identifying the candidate genes for crucial traits. The lack of the whole genome sequence in a majority of the ornamentals is a limitation to deciphering the molecular and regulatory networks controlling a trait. Potential drawbacks for CRISPR/Cas9 include off-target effects, which occur when a Cas9 nuclease cleaves DNA sequences in nontargeted sites resulting in unintended genetic modifications; this indicates the need to minimize off-target effects [42] (Zhao and Wolt, 2017). In addition, off-targets often occur in the non-protein-coding regions, which leads to modifications in gene expression and regulatory networks. Hence, the identification of potential nontarget off-targets in noncoding regions is essential, but is highly difficult because of their huge number. Although bioinformatics tools detect these off-target sites, only lower accuracy is possible for the noncoding regions [43] (Tycko et al., 2019). Successful genome editing requires an efficient delivery of Cas9 components into plant cells, which depends on effective transformation protocols. However, plant transformation is challenging in some of the ornamental crops as they are recalcitrant, which suggests the necessity of developing a genotype-flexible plant transformation system [44] (Kausch et al., 2019). Stable inheritance of gene-edited traits with reliable transfer through subsequent generations is crucial for a genetically modified plant. Enhancing the inheritance and segregation requires efficient screening and selection of gene-edited lines [45] (Mao et al., 2019). Major challenges for genetic engineering include the complex genomes and recalcitrance of ornamental plants. Nevertheless, both genome editing and genetic engineering offer potential platforms for developing novel varieties with improved traits (Figure 1).

## 3. Importance of Improving Ornamental Attributes

The ornamental value of a plant is imparted by various aesthetic attributes such as the vibrant colors and attractive shapes of flowers, fruits, leaves, floral fragrance, plant architecture, variegation and leaf texture. Apart from the aesthetic value, these traits often possess medicinal and nutritional value in some ornamentals like chrysanthemum. Aesthetic appearance is crucial to the economic value of the ornamental plant because the customer’s choice depends on the visual quality. In addition, consumer preference is highly dynamic and changes rapidly, and the market constantly requires novel traits. Thus, ensuring the visual quality of these traits and introducing novel varieties with improved ornamental attributes have been the major objectives of breeders to sustain the dynamic ornamental market. Since ornamental plants are grown for their aesthetic value, ornamental plant breeding is mainly aimed at visual characteristics such as floral traits and plant architecture. However, other traits like longer shelf life, regulation of flowering time and resistance to both biotic and abiotic stresses also constitute crucial characteristics for obtaining higher yields and visually healthy plants. Nevertheless, these traits are usually considered as secondary and are monitored only as additional characteristics during the later stage of the breeding line selection process. Hence, the breeding research to improve these traits is scanty in ornamental plants [10]. The major hurdle most of the ornamental plants encounter is sexual hybridization because of their higher heterozygosity, sterility, higher chromosome number and longer life spans. Important ornamentals such as carnations are self-fertile and unable to generate seeds, the huge genome sizes in chrysanthemums and lilies make genome mining harder, and the life cycle of anthuriums and some orchids is about 3 years, which means a longer time period is required to develop a cultivar [46,47,48]. Cross-breeding has been beneficial in developing various novel cultivars with morphological variations. However, since this breeding method mainly depends on the phenotype to select the elite parents, it is laborious for traits such as stress resistance [49]. Also, crucial traits such as a blue-colored flower, which are naturally absent in chrysanthemums and roses, are not possible to produce via conventional breeding. Although molecular breeding significantly improved breeding efficacy, it has been limited by various hurdles in ornamental plants such as huge and complex genomes, smaller gene pools, etc. [50]. Alternatively, both genetic engineering and genome editing technologies have been proving to be promising tools. Apart from the floral traits, other important ornamental attributes such as plant architecture, postharvest vase life and biotic and abiotic tolerance are being addressed with genetic engineering [51]. Genome editing in ornamentals is still at a slower pace owing to their complex genomes, and its application has been reported for few important floral traits [52]. The potential of genetic engineering and genome editing in the enhancement of various floral traits in ornamental plants is discussed in the following sections.

## 4. Applications of Genetic Engineering and Genome Editing to Improve Floral Traits in Ornamentals

### 4.1. Floral Color

**Figure 2:**
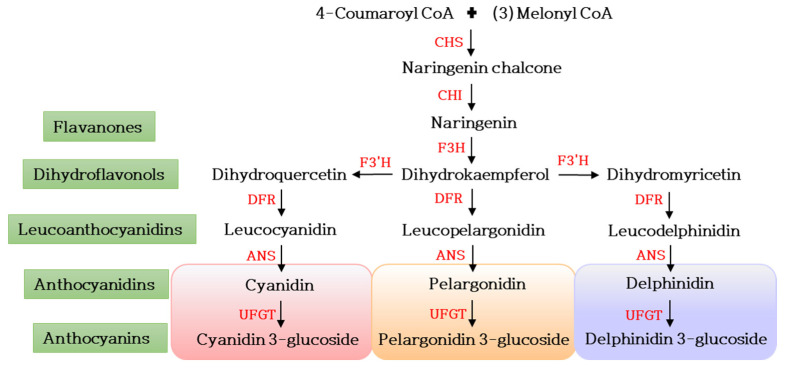
Schematic representation of anthocyanin biosynthesis pathway. Enzymes involved in the anthocyanin biosynthesis are shown in red. CHS—chalcone synthase; CHI—chalcone isomerase; F3H—flavone 3-hydroxylase; hydroxylase; F3′H—flavonoid 3′-hydroxylase; F3′5′H—flavonoid 3′5′-hydroxylase; DFR—dihydroflavonol 4-reductase; ANS—anthocyanidin synthase; UFGT—anthocyanidin synthase. The colored anthocyanin background represents the respective color.

Floral hue represents the most alluring and beautiful attribute of ornamental plants that also has commercial significance. Apart from the aesthetic value, petal color is vital in pollinator attraction for reproduction. It is a biologically important trait, with the pigments playing a crucial role in protection from photo-oxidative damage, imparting biotic and abiotic stress resistance [53]. Also, floral color is an extensively studied trait in ornamentals. Petal coloration is usually attributed to plant pigments such as flavonoids, carotenoids, anthocyanins, betalains and α and β chlorophylls. Chlorophylls impart a green color and carotenoids are mainly responsible for yellow, orange and red colors. Anthocyanins are classified into cyanidins, pelargonidins, delphinidins, petunidins, malvidins and peonidins. Cyanidins, pelargonidins and delphinidins are the major anthocyanins, which are responsible for a diverse range of colors, from orange and red to purple and blue. Anthoxanthins produce white and light yellow flowers [54] (Figure 2). Various ornamental plants have been genetically engineered for flower color modifications through targeting floral pigments such as anthocyanins, carotenoids and betalains [55]. The transgenic petunia was the first ornamental plant that was flower-color-modified via overexpressing the *Zea mays A1* gene that encodes dihydroflavonol reductase, which is absent in petunias, resulting in a pelargonidin-expressing orange-colored flower [56]. Modification of floral color has been achieved by targeting the key biosynthetic genes either by overexpression, by downregulation or by silencing the gene. Sequence-specific degradation via post-transcriptional regulation of the *CHS* gene resulted in a star-type pigmentation pattern in the corollas of petunias [57]. Downregulation of *CHS* showed a total pigmentation loss leading to white-colored flowers in chrysanthemum and petunia [58]. It has been reported that a lack of *CHI* activity is required for the formation of yellow color, and a *CHI*-suppression by *RNAi* in tobacco resulted in reduced pigmentation and the flower color changed to yellow in some of the transgenic lines [59]. Boase et al. [60] reported the first genetic modification of cyclamen, which was achieved by suppressing the *F3′5′H* gene, leading to a shift in the floral color from purple to red/pink in the transgenic lines. A transgenic expression of gerbera *DFR* and suppression of the *F3′5′H* gene resulted in a shift in anthocyanin biosynthesis from delphinidin to pelargonidin accumulation in the *Osteospermum hybrida* transgenic lines [61]. Genetic transformation of *Viola F3′5′H* along with the *DFR* gene from *Iris x hollandica* produced a new blue phenotype in transgenic roses [62]. Transgenic gerbera plants overexpressing *GMYB10* induced cyanidin synthesis, leading to increased accumulations of pigments [63]. Overexpression of *F3′5′H* from *Phalaenopsis* in the *Lilium* oriental ‘Sorbonne’ turned the flower color to pale purple from pink, while a co-expression of *Ph F3′5′H* and *HyDFR* resulted in dark purple-colored flowers [64]. In chrysanthemum, since the delphinidin pathway is absent and blue-colored flowers do not exist naturally, He et al. [65] attempted to shift the anthocyanin biosynthesis from the cyanidin to the delphinidin pathway by overexpressing *Senecio cruentus F3′5′H* and downregulating the *F3′H* gene, which was, however, not successful, and resulted in the production of bright red flowers due to the overaccumulation of cyanidins. The delphinidin pathway was engineered via expressing a chimeric pansy *F3′5′H* under floral specific promoters, and in another study, the chrysanthemum *F3H* promoter-driven *alcohol dehydrogenase* (*ADH*) translational enhancer-fused Campanula *F3′5′H* was co-expressed, resulting in violet/blue flowers in both the studies [66,67]. In a further attempt, Noda et al. [68] achieved a true-blue-flowered chrysanthemum through the co-expression of the *uridine diphosphate (UDP)-glucose–anthocyanin 3′,5′-O-glucosyltransferase* gene from a butterfly pea and a Canterbury bell’s *F3′5′H*. The simultaneous transient expression of *chalcone 4′-O-glucosyltransferase* (*4′CGT*) and *aureusidin synthase* (*AS1*) genes, bypassing the silencing of anthocyanin biosynthetic pathway genes, resulted in a change in floral color from white to yellow in transgenic African violet petals [69]. A recent overexpression of *PhCHS5* and *PhF3′5′H* genes in petunia and phalaenopsis resulted in deeper floral lip color in both transgenic plants, suggesting the relevance of these genes in phalaenopsis breeding for novel colors [70]. Overexpression of *RcMYB1* transcription factor substantially improved the accumulation of anthocyanins in the white petals of transgenic rose lines [71]. Similarly, co-overexpression of *F3′5′H* from *Viola tricolor* and *Rosa hybrida NHX* genes in white rose lines resulted in a color change from white to red-purple [72].

With the advances in NGS technologies, genome sequence information with accuracy is being developed for various crops. In recent years, the genome sequences of several ornamental plants have been reported. Also, genome editing systems, especially CRISPR/Cas9, offer tremendous possibilities for the breeding of ornamentals by improving valuable floral traits. However, some of the polyploid ornamentals like chrysanthemum and roses need a highly efficient gene editing system. Modification of flower color is currently the most studied trait for the application of genome editing. CRISPR/Cas9 was first applied for flower color modification in *Ipomea nil* via modification of the expression of the *DFR* gene [73]. Further, mutant *Ipomea nil* plants bearing pale yellow petals were produced by CRISPR/Cas9 targeting the *carotenoid cleavage dioxygenase 4* (*CCD4*) gene [74]. A mutation of the *F3H* gene by CRISPR/Cas9 in *Torenia fournieri* led to the flower color shifting from pale blue to white [75]. *Phytoene desaturase* (*PDS*) from carotenoid biosynthesis was mutated, with the resulting mutant *Lilium* lines exhibiting albino, albino-green and pale yellow pigmentation patterns in the flower [76]. CRISPR/Cas9 editing of *glutathione transferase 1* (*GST1*) in Japanese gentian flowers produced mutants with white and mild blue phenotypes [77]. The duplicated genes *F3HA* and *F3HB* were targeted simultaneously and transformed into petunia protoplasts. Among the resultant plants generated from the protoplasts, only one plant showed a color change to light purplish pink from the original purple color [78]. Cas9-mediated mutation of an R2R3-MYB transcription factor, *DPL*, resulted in the vein-associated absence of an anthocyanin pattern on the bud, and as reported earlier, it did not show corolla tube venation. However, CRISPR/Cas9 mutations in the *AN4* gene caused an absence of corolla tube venation, suggesting that *AN4* is a key regulator of the corolla tube venation trait [79].

### 4.2. Floral Scent

Along with floral color, floral scent is a major commercial ornamental trait that also possesses aesthetic and biological significance for pollinator attraction and protection against pathogens. ‘Flowers with fragrance’ are in high demand and floral scent compounds are used in perfumes, cosmetics, dietary fields and medicine. Flowers emit volatile organic compounds (VOCs), and these components of floral scent primarily belong to three groups, namely terpenoids, phenylpropanoids/benzenoids and fatty acid derivatives [80]. Different plant species form specific scents due to the production of various combinations of these VOCs. The engineering of floral scent is a relatively new area and the molecular and biochemical studies on floral fragrance are comparatively few in number. The complex hereditary patterns of floral scent make breeding harder for this trait. Studies in recent years allowed the characterization of floral volatiles and the genes that regulate fragrance in different ornamental plants. Marketed cultivars of roses and carnations bred for cut flowers usually do not possess fragrance due to the importance of selecting other traits. However, efforts to induce floral scent via genetic engineering have produced some ornamental plants with improved fragrance. In order to induce scent in lisianthus, the *benzyl alcohol acetyl transferase* (*BEAT*) gene from *C. breweri* was transformed for the production of benzyl acetate, which is a crucial component of scent. VOCs, including benzyl acetate, were produced in transgenic flowers in the presence of an alcoholic substrate, suggesting that the alcoholic substrate is essential for scent production in transgenic lisianthus flowers [81]. Similarly, transgenic carnation plants overexpressing the *C. breweri linalool synthase* (*lis)* gene produced crucial floral scent compounds such as linalool and its derivatives. However, linalool emission in transgenic carnation flowers could not produce human olfaction-detectable floral scent [82]. Expression of the *Arabidopsis thaliana Anthocyanin Pigment1* (*Pap1*) MYB transcription factor in petunia increased the production of phenylpropanoid/benzenoid compounds in transgenic petunia flowers [83]. Also, genetic transformation of *Pap1* in roses improved terpenoid and phenylpropanoid VOCs in transgenic flowers [84]. Linalool is a major component of VOCs with a sweet fragrance that performs a crucial role in a plant’s defense. Expression of the *linalool/nerolidol synthase* (*FaNES1*) gene in chrysanthemum enhanced the production of linalool and derivatives, which increased scent; this initially attracted western flower thrips, but they avoided the flowers later due to the bad taste of these VOCs [85]. Although floral scent is an important ornamental attribute, inadequate understanding of scent metabolic pathways hinders the genetic engineering and genome editing of floral scent-related traits in ornamental plants.

### 4.3. Flower Longevity

**Figure 3:**
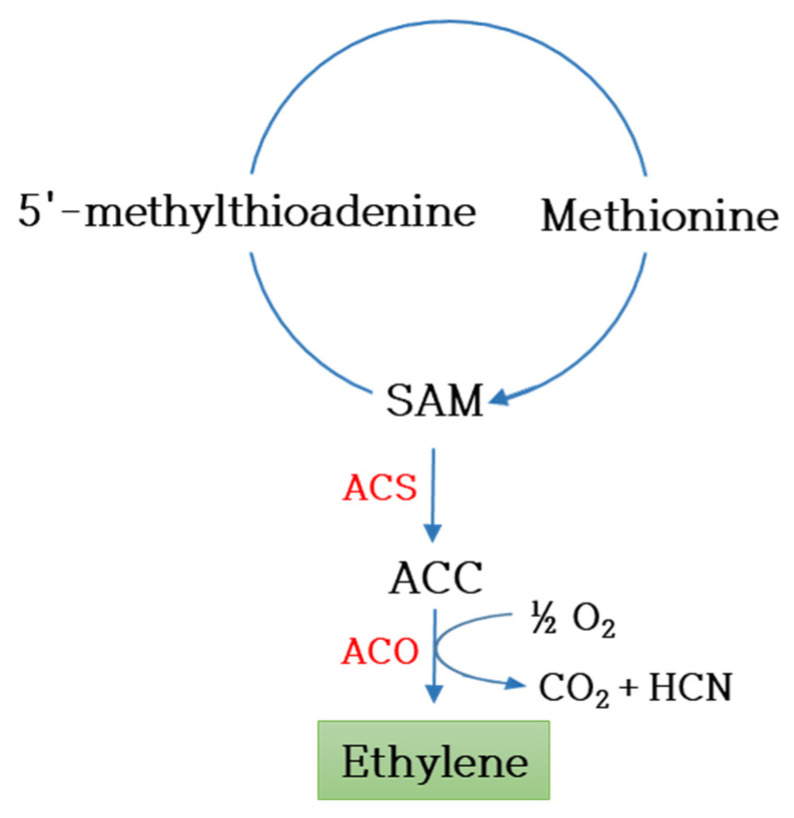
Schematic representation of ethylene biosynthesis pathway. ACS—ACC synthase; ACO—ACC oxidase.

After being harvested, cut flowers are transported and distributed without roots, during which time their storage and quality maintenance are highly difficult. Since flowers are short-lived, it is important to reduce the postharvest losses during export. Quantitative characteristics like flower size, cut flower weight, number of leaves and number of flowers are also crucial in determining the cut flower quality. Hence, senescence, loss of organs and other postharvest damages need to be addressed with molecular and biotechnological tools apart from the postharvest chemical treatments to enhance the cut flower’s shelf life [86]. Shelf life of a few weeks, longer vase life, resistance to bacterial infection during storage and ethylene-induced senescence factors are the major target traits for preventing postharvest losses [87]. The plant hormone ethylene is responsible for senescence and the inhibition of ethylene biosynthetic genes, which increases the vase life (Figure 3). Various ornamental plants have been genetically manipulated to inhibit ethylene-induced senescence by blocking ethylene perception and biosynthesis. The transformation of the mutated ethylene receptor gene *mDG-ERS1* in chrysanthemum revealed its ability to inhibit the sensitivity to ethylene, and the transgenic chrysanthemum expressing mutated *mDG-ERS1* (*etr1-4*) exhibited reduced leaf senescence [88,89]. Similarly, mutated *etr1-1* from *Arabidopsis thaliana* suppressed ethylene susceptibility in various transgenic ornamental plants including carnations, campanulas, orchids and pelargoniums [90,91,92,93,94]. Delayed flower senescence associated with lower ethylene production was observed in transgenic carnations with the sense ACC oxidase gene [95]. Alternatively, increased cytokinin levels induce delayed senescence, which was evident in a transgenic petunia and miniature rose overexpressing *P_SAG12_-IPT*, leading to the regulation of cytokinin pathways resulting in reduced ethylene sensitivity and delayed senescence [96,97].

*EPHIMERAL1 (EPH1)* is an NAC transcription factor that plays a key role in the regulation of senescence. CRISPR/Cas9 gene editing of *EPH1* led to a target site mutation in the edited T_0_ lines of Japanese morning glory, and the T_1_ lines showed delayed petal senescence [98]. CRISPR/Cas9 was applied to edit ethylene biosynthesis enzyme coding gene *1-aminocyclopropane-1-carboxylate oxidase1* (*PhACO1*) in the petunia cultivar ‘Mirage Rose’, and the flowers of the transgenic petunias showed delayed senescence associated with low ethylene production [99]. In contrast, CRISPR/Cas9-mediated gene editing of *Autophagy gene 6* (*PhATG6*) in petunia increased ethylene production and senescence-related gene expression, leading to accelerated petal senescence [100]. A recent knock-out mutant of rose for the *ETHYLENE INSENSITIVE2* (*RhEIN2*) gene, which is a key player in ethylene signaling, showed ethylene sensitivity and blocking of flower opening in the rose [101].

### 4.4. Floral Anatomy

The development of novel flower shapes and patterns is essential for ornamental plants to maintain their market value, and hence the ‘floral figure’ is an important breeding goal. However, molecular mechanisms responsible for the development of floral patterns remain largely unexplored. The differentiating cells of the floral meristem construct the floral architectural beauty in concentric whorls. Flower shape was modified in chrysanthemum via the suppression of the *AGAMOUS* gene, which resulted in the change of the gynoecium and androecium to corolla-like tissues, thus altering the floral shape [102]. Ectopic expression of *PttKN1* in transgenic carnations showed pleiotropic morphological alteration and modification in the phyllotaxis [103]. Transgenic lisianthus flowers overexpressing the *MADS1-M* gene from lily exhibited an altered floral structure with the change of the second whorl of petals into sepal-like structures and a visible deformation of third whorl stamens [104]. Overexpression of *GhSOC1*, a paralog of *AtSOC1*, caused flower shape modification with reduced epidermal cell size in ray petals and a loss of floral distinctiveness [105]. Overexpression of *CmCYC2c* in chrysanthemum increased the length of ray florets and flower number per plant, but no significant change in the floral shape was observed [106]. The chrysanthemum polarity homologous gene, *CmYAB1*, when expressed ectopically, reduced the petal curvature of flat petals, and the transgenic plants showed round pompon-like inflorescence [107]. Transgenic phalaenopsis overexpressing *PhCHS5* and/or *PhF3′5′H* exhibited additional phenotypes of more petals, labial petals and branches apart from the enhanced floral color [70].

Su et al. [108] developed Cas9 loss of function *TfRAD1* (*RADIALIS1*) mutant lines of *Torenia fournieri* which had a similar phenotype to that of *TfCYC2* (*CYCLOIDEA2*)-RNAi lines with a violet color pattern on dorsal petals and ventralized later petals. Cas9 editing of the *piSSK1* gene of the petunia SCF-SLD complex to test *piSSK1*’s effect on self-incompatibility resulted in the loss of piSSK1 in pollen grains, leading to growth inhibition of pollen tubes [109]. CRISPR/Cas9-mediated mutations in the miR172 target sequence in the TOE-type genes *PETALOSA* (*PET)* resulted in an increased number of petaloid stamens in gene-edited tobacco plants, which is similar to the effects of mutations that naturally occur in the double-flower phenotype of petunia, carnation and *R. rugosa* [110]. Recently, Nishihara et al. [111] used the CRISPR/Cas9 system to enhance double-flowered genetic resources in gentian, which only possesses a single-flower type naturally. Genome editing of the *AGAMOUS* (*AG*) floral homeotic gene (*AG1*) successfully produced double-flower-type gentian plants and further produced transgene-free genome-edited null segregant gentian plants.

### 4.5. Flowering Time and Development

Flowering time represents the number of days to initial flowering from the planting day. Flowering time is an important trait that determines commercial success. The crucial event of a plant’s transition from the vegetative to the reproductive stage is induced by a series of endogenous and environmental cues [112]. Establishment of a floral regulation system is crucial for economic gains, and commercial-level production of plants requires precise flowering time. Also, early flowering enables the availability of flowers in short periods and reduces the production cost, making the crop commercially beneficial. The development of early flowering cultivars to reduce the flowering time and of cultivars that can flower during long days is the key breeding goal in ornamentals. Molecular tools have been applied to regulate flowering, and various ornamental cultivars with specific flowering times have been developed. The transgenic chrysanthemum overexpressing *AP1* gene, one of the MADS-box genes that are crucial for regulating flowering time and floral organ development, exhibited an early bud initiation of about 14 days earlier than the control plants during long days. In addition, the transgenic lines showed early inflorescence opening and color patterns compared with control plants [113]. Flowering locus overexpression or suppression of miRNA159 induced late or early flowering in transgenic gloxinia plants. Expression levels of miRNA159 influenced up- or downregulation of *SsGAMYB* during floral development, suggesting mir159-mediated *GAMYB* expression plays a key role in regulating the flowering period [114]. The flowering locus T-like (FTL) paralog *CsFTL3* from *Chrysanthemum seticuspe* has been identified to be involved in the photoperiodic regulation of flowering. Overexpression of *CsFTL3* constitutively in chrysanthemum led to floral bud development under long-day conditions [115]. Sucrose treatment-induced *CmFTL3* played an active role in floral transition and regulation of photoperiodic flowering in short-day conditions [116]. Nevertheless, *CmFTL1* showed a lower florigenic activity when expressed constitutively in a short-day chrysanthemum cultivar, ‘Jinba’ [117]. Studies showed that the overexpression of FT orthologues from *Lilium longiflorum* (*LlFT*) and *Tulipa gesneriana* (*TgFT3*) resulted in early flowering in *Arabisopsis thaliana*, and the overexpression of *LlFT* in lily caused consistency in early flowering [118]. Transgenic chrysanthemum expressing *TERMINAL FLOWER1* (*CmTFL1a*) in the chrysanthemum cultivar ‘Jinba’ delayed the transition from the vegetative to the reproductive phase [119]. Overexpression of the BBX family zinc finger transcription factor, *CmBBX8*, a *CmFTL* activator, accelerated the flowering by 20 days compared with control plants, and its suppression caused a delay in flowering of 15 days [120], whereas suppression of *CmBBX24* in transgenic chrysanthemum lines resulted in early flowering compared with wild-type lines [121]. In contrast, constitutive overexpression of *CmBBX29* in transgenic *Arabidopsis* delayed flowering through the suppression of flowering genes [122]. Overexpression of an R2R3 *MYB* transcription factor in transgenic chrysanthemum, *CmMYB2*, accelerated flowering and its downregulation delayed flowering, both of which were associated with the variation in gibberellin synthesis, suggesting an interaction with *CmBBX24*, which regulates gibberellin-mediated flowering [123]. Ectopic expression of a transcription factor from *Arabidopsis*, *LEAFY* (*AtLFY*) in *Tricyrtis* sp., produced early flowering and dwarf transgenic plants, suggesting the possibility of developing dwarf and early flowering plants with *LFY* genes [124]. A recent study identified four *SEPALLATA*-like genes from *Cymbidium sinense*, *CsSEP1*, *CsSEP2*, *CsSEP3* and *CsCEP4*, and characterized the genes through their transformation of *Arabidopsis*. Transgenic *Arabidopsis* expressing these four *CsSEP* genes exhibited the early flowering phenotype. Early flowering was associated with expression of endogenous flowering-related genes, suggesting that *CsSEP* regulates flowering by inducing downstream flowering genes [125]. Various genes that have been manipulated with genetic engineering to improve different floral traits are briefly listed in Table 2.

CRISPR/Cas9 was successfully used to generate multiple mutants of *Phalaenopsis equestris MADS* genes. *MADS*-null mutants of phalaenopsis suggest the potential of this application for gene family studies in plants with a long juvenile period [126]. Liu et al. [127] identified *TFL1* homologues in *Chrysanthemum indicum*, *CiTFL1a* and *CiTFL1b*, and mutants of these genes were generated using CRISPR/Cas9. The mutant plants exhibited different degrees of early flowering and among the two types of mutants, *Citfla* mutants showed the earliest flowering phenotype. *Phalaenopsis amabilis* is an orchid with a long vegetative period, and to shorten this period to induce flowering, the early flowering mutant gene known as the *Gibberellic Acid Insensitive* (*GAI*) gene has been identified for the CRISPR/Cas9 editing system to accelerate flowering [128]. Various gene-edited ornamental plants using CRISPR/Cas9 to date are listed in Table 3.

## 5. Conclusions and Future Prospects

The global flower and ornamental plant market is valued at USD 43.91 billion in 2023 from USD 40.25 billion in 2022, which is forecasted to increase further in subsequent years. Growing demand for ornamental plants and cut flowers requires the incessant development and introduction of novel and improved cultivars. Adaption and application of emerging tools and technologies are essential to overcome limitations in order to improve and introduce highly desired traits. Although, over the years, a wide range of cultivars with beneficial traits have been developed, potential tools are needed to surpass the challenges of complex genetic backgrounds, longer life cycles, polyploidy and self-incompatibility to enhance the breeding efficiency. Traditional breeding methods via recombination and hybridization are laborious, imprecise, time-consuming and unpredictable. To overcome these intrinsic barriers in conventional breeding, genetic manipulation has been applied as an alternative potential tool. Genetic engineering has several advantages over traditional breeding, like the introduction or manipulation of specific traits without alteration of the endogenous traits. Numerous beneficial traits have been improved and developed using transgenic technology, and these are often impossible to achieve with conventional breeding, such as the blue-colored flower in chrysanthemums and roses. Novel transgenic varieties therefore offer potential gains to both growers and consumers. Nevertheless, genetic engineering also has limitations, and one of the major constraints is the regulatory approval to commercialize the developed transgenic plants. Precise and rapid site-directed approaches to modify the genes are a promising alternative for improving traits. Genome editing tools, including CRISPR/Cas9, are a breakthrough technology that has revolutionized functional genomics and applied crop breeding. Their nature of higher specificity, simplicity, productivity and multiplexing flexibility makes them desirable tools. The application of genome editing is beneficial in ornamental plants that are characterized by various challenges which limit their conventional breeding. Also, DNA-free editing methods are strongly required to achieve non-transgenic edited plants. Although genome editing is still in its infancy in ornamental plants, it has become a popular molecular tool of choice for functional genomics and trait improvement studies. Despite its potential benefits, the applicability and efficiency of gene editing encounter limitations in ornamental plants such as recalcitrancy in several ornamental plants and low efficiency of gene editing due to the complex genetic background of the target. Efficient tools for surpassing these barriers and research on functional genomics and genome engineering are required for the reliable application of genome editing and genetic engineering in ornamental plants. Future goals for the improvement of ornamental plants via both genome editing and genetic engineering require deeper deciphering of the molecular networks regulating the traits to identify and expand the gene pool availability. Cas codon optimization allows for identifying highly specific and efficient promoters and minimizing off-target modifications by bioinformatics tools for increased editing efficiency. The development of genotype-independent regeneration protocols and strategies for stable inheritance of the target engineered gene through efficient genotyping and screening methods is crucial for both genome editing and genetic engineering. Future studies on effective application and implementation of these cutting-edge futuristic tools would revolutionize the ornamental horticulture industry.

## Figures and Tables

**Table 1:** Trend in the turnover of top 15 ornamental plants as reported by Royal FloraHolland auction in 2022.

S. No	Ornamental Plant	Turnover (Million Euros)
Cut Flowers		
1	Rose	694
2	Chrysanthemum	369
3	Tulip	257
4	Lily	155
5	Eustoma russellianum	149
House Plants		
6	Phalaenopsis	402
7	Arrangements	69
8	Anthurium	64
9	Kalanchoe	64
10	Rose	59
Garden Plants		
11	Helleborus	25
12	Hydrangea	24
13	Lavandula	21
14	Carnation	20
15	Bedding plants	20

**Table 2:** List of studies involved in the manipulation of various ornamental attributes through the genetic engineering of different genes resulting in improved traits.

Floral Attribute	Genetic Engineering				
	Gene	Source	Target Plant	Resulting Trait	Ref.
Floral Color	*A1*	Zea mays	Petunia	orange-colored flower	[52]
	*CHS*	Petunia	Petunia	star-type pigmentation pattern in corolla	[53]
	*CHS*	Petunia, chrysanthemum	Petunia, chrysanthemum	pigmentation loss and white-colored flower	[54]
	*CHI*	Tobacco	Tobacco	floral color shift to yellow	[55]
	*F3′5′H*	Cyclamen	Cyclamen	floral color shift from purple to red/pink	[56]
	*DFR*	Gerbera	*Osteospermum hybrida*	shift from delphinidin to pelargonidin	[57]
	*F3′5′H*, *DFR*	Viola, *Iris x hollandica*	Rose	blue-colored flower phenotype	[58]
	*GMYB10*	Gerbera	Gerbera	cyanidin synthesis and increased pigmentation	[59]
	*F3′5′H*	Phalaenopsis	Lilium	floral color shift from pink to purple	[60]
	*F3′5′H*	*Senecio cruentus*	Chrysanthemum	bright red flower	[61]
	*F3′5′H*	Pansy	Chrysanthemum	violet/blue flower	[62]
	*ADH+* *F3′5′H*	Campanula	Chrysanthemum	violet/blue flower	[63]
	*F3′5′H*	Canterbury bells	Chrysanthemum	true-blue-colored flower	[64]
	*4′CGT+AS1*	*A. majus*	African violet	floral color shift from white to yellow	[65]
	*PhCHS* *PhF3′5′H*	*Phalaenopsis*	Phalaenopsis, petunia	deeper floral lip color	[66]
	*RcMYB1*	Rose	Rose, tobacco	increased anthocyanins in white petals	[67]
	*F3′5′H+NHX*	Viola, rose	Rose	floral color shift from white to red-purple	[68]
Floral Scent	*BEAT*	*C. breweri*	Lisianthus	floral scent	[77]
	*lis*	*C. breweri*	Carnation	floral scent	[78]
	*Pap1*	*Arabidopsis*	Petunia	floral scent	[79]
	*Pap1*	*Arabidopsis*	Rose	floral scent	[80]
	*FaNES1*	Strawberry	Chrysanthemum	floral scent	[81]
Floral Longevity	Mutated *etr1-4*	Chrysanthemum	Chrysanthemum	reduced leaf senescence	[85]
	Mutated *etr1-1*	*Arabidopsis*	Carnation, campanula, orchids	suppressed ethylene susceptibility	[86,87,88,89,90]
	*ACC oxidase*	Carnation	Carnation	lower ethylene production	[91]
	*P_SAG12_-IPT*	*A. tumefaciens*	Petunia and rose	delayed senescence	[92,93]
Floral Anatomy	*AGAMOUS*	Chrysanthemum	Chrysanthemum	floral shape change	[98]
	*PttKN1*	Hybrid aspen	Carnation	modification of phyllotaxis	[99]
	*MADS1-M*	Lily	Lisianthus	altered floral structure	[100]
	*GhSOC1*	Gerbera	Gerbera	loss of floral distinctiveness	[101]
	*CmCYC2c*	Chrysanthemum	Chrysanthemum	increased ray floret length	[102]
	*CmYAB1*	Chrysanthemum	Chrysanthemum	reduced petal curvature and pompon flower	[103]
	*PhCHS5,* *Ph F3′5′H*	Phalaenopsis	Phalaenopsis	increased petals, labial petals	[66]
Flowering Time	*AP1*	*Asteraceae*	Chrysanthemum	early flowering	[109]
	*miR159*	Gloxinia	Gloxinia	flowering time regulation	[110]
	*CsFTL3*	*Chrysanthemum seticuspe*	Chrysanthemum	floral bud development	[111]
	*LlFT*	*Lilium longiflorum*	Lily	early flowering	[114]
	*CmTFL1a*	Chrysanthemum	Chrysanthemum	delayed flowering	[115]
	*CmBBX8*	Chrysanthemum	Chrysanthemum	early flowering	[116]
	*CmBBX24*	Chrysanthemum	Chrysanthemum	early flowering	[117]
	*CmBBX29*	Chrysanthemum	Arabidopsis	delayed flowering	[118]
	*CmMYB2*	Chrysanthemum	Chrysanthemum	early flowering	[119]
	*AtLFY*	Arabidopsis	Tricyrtis sp.	early flowering	[120]
	*CsSEP1,2,3,4*	*Cymbidium sinense*	Arabidopsis	early flowering	[121]

**Table 3:** List of genome editing studies with CRISPR/Cas9 in various ornamental plants to manipulate different floral traits.

Ornamental Attribute	Genome Editing (CRISPR/Cas9)			
	Gene	Target Plant	Resulting Trait	Reference
Floral Color	*DFR*	*Ipomea nil*	floral color modification	[69]
	*CCD4*	*Ipomea nil*	pale yellow-colored petals	[70]
	*F3H*	*Torenia fournieri*	floral color shift from pale blue to white	[71]
	*PDS*	Lily	albino, albino-green and pale yellow flower pigmentation	[72]
	*GST1*	Japanese gentian	white and mild blue floral phenotype	[73]
	*F3HA, F3HB*	Petunia	color shift from purple to purplish pink	[74]
	*DPL*	Petunia	vein-associated absence of anthocyanin pattern	[75]
	*AN4*	Petunia	absence of corolla tube venation	[75]
Floral Longevity	*EPH1*	Japanese morning glory	delayed petal senescence	[94]
	*PhACO1*	Petunia	delayed senescence	[95]
	*PhATG6*	Petunia	accelerated senescence	[96]
	*RhEIN2*	Rose	ethylene sensitivity	[97]
Floral Anatomy	*TfRAD1*	*Torenia fournieri*	violet color pattern on dorsal petals and ventralized later petals	[104]
	*piSSK1*	Petunia	growth inhibition of pollen tubes	[105]
	*PET*	Tobacco	double-flower phenotype	[106]
	*AG1*	Gentian	double-flower phenotype	[107]
Flowering Time and Development	*MADS*	Phalaenopsis	gene editing efficiency in flowering time	[122]
	*CfTFL1a, CiTFL1b*	*Chrysanthemum indicum*	early flowering	[123]
	*GAI*	*Phalaenopsis amabilis*	early flowering	[124]

## Data Availability

All the data are contained within the article.
